# Stability and Reactivity of Cyclopentane Nucleoside Analogs in 98% *w*/*w* Sulfuric Acid

**DOI:** 10.3390/molecules31061003

**Published:** 2026-03-17

**Authors:** Sara Seager, Maxwell D. Seager, Ton Visser, Nittert Marinus, Mael Poizat, Jim van Wiltenburg, Martin Poelert, Janusz J. Petkowski

**Affiliations:** 1 Department of Earth, Atmospheric and Planetary Sciences, Massachusetts Institute of Technology, Cambridge, MA 02139, USA; 2 Department of Physics, Massachusetts Institute of Technology, Cambridge, MA 02139, USA; 3 Department of Aeronautics and Astronautics, Massachusetts Institute of Technology, Cambridge, MA 02139, USA; 4 Nanoplanet Consulting, Concord, MA 01742, USA; 5 Department of Chemistry and Biochemistry, Worcester Polytechnic Institute, Worcester, MA 01609, USA; 6 Symeres Netherlands BV, Kadijk 3, 9747 AT Groningen, The Netherlands; 7 Faculty of Environmental Engineering, Wroclaw University of Science and Technology, 50-370 Wroclaw, Poland; 8 JJ Scientific, 02-792 Warsaw, Poland

**Keywords:** carbocyclic nucleosides, cyclopentane nucleoside analogs, concentrated sulfuric acid, ^13^C NMR, ^1^H NMR

## Abstract

We synthesized seven carbocyclic nucleoside analogs featuring a cyclopentane ring in place of the (deoxy)ribose sugar, which serves as a linker in DNA/RNA nucleosides. We assessed the stability of cyclopentane nucleosides in 98% *w*/*w* sulfuric acid at room temperature via ^1^H and ^13^C NMR spectroscopy. We observe that adenine (**A1**, **A4**), guanine (**G1**) and thymine (**T1**) cyclopentane nucleoside analogs remain stable for at least two weeks at room temperature, with only minor (~4%) degradation in **A1**. In contrast, the cytosine analog (**C1**) rapidly degrades to release a soluble cytosine. Methyl-substituted adenine analogs mimicking polymer backbone attachments at positions prone to tertiary carbocation formation (**A2**, **A3**) prove unstable and release soluble adenine. Only the 3,3-dimethylcyclopentyl adenine analog (**A4**) exhibits sufficient stability. Our findings reveal that cyclopentane serves as a viable stable linker in concentrated sulfuric acid for select nucleic acid bases, provided that the backbone connections avoid tertiary carbons susceptible to carbocation-mediated cleavage. We thus identify one potential key structural feature for engineering examples of genetic-like polymers that could potentially persist in Venus’s concentrated sulfuric acid cloud environment.

## 1. Introduction

Organic chemistry in concentrated sulfuric acid is rarely studied despite its surprising richness. Recent studies, building on isolated reports from decades ago (e.g., [1,2,3,4,5,6,7,8]), have renewed the interest in this area of organic chemistry, showing that complex organic molecules can stably persist in this aggressive solvent.

The resurgence of interest in organic chemistry of concentrated sulfuric acid stems from speculations about possible life in Venus’s atmosphere—not on the scorching 700 K surface but within the sulfuric acid clouds at 48–60 km altitude where temperatures resemble those on Earth’s surface (e.g., [9,10,11,12,13,14,15,16,17,18,19]). Although complex organic chemistry does not equate to life, its presence in a planetary environment provides an essential prerequisite for the possibility of life [20].

Recently, Spacek and Benner have demonstrated that diverse organic reactions spontaneously emerge in concentrated sulfuric acid from simple starting materials like formaldehyde or carbon monoxide [21,22,23]. Our team showed the stability of simple organic chemical building blocks, including nucleic acid bases [24,25], amino acids [26] and dipeptides [27,28]. We have also shown that simple lipids are not only stable but spontaneously form lipid vesicles, micelles and other complex membranous structures in concentrated sulfuric acid at room temperature [29].

This recent work shows promise, yet life requires far more sophisticated molecular structures—particularly complex polymers—to perform biological functions. In particular, the need for genetic polymers with functional and structural properties similar to RNA and DNA seems to be the universal requirement for life, regardless of life’s underlying chemistry [20,30]. Therefore, identifying genetic polymers that resist degradation in concentrated sulfuric acid becomes a critical step to study the possibility of life in environments where sulfuric acid is a dominant liquid.

**Figure 1:**
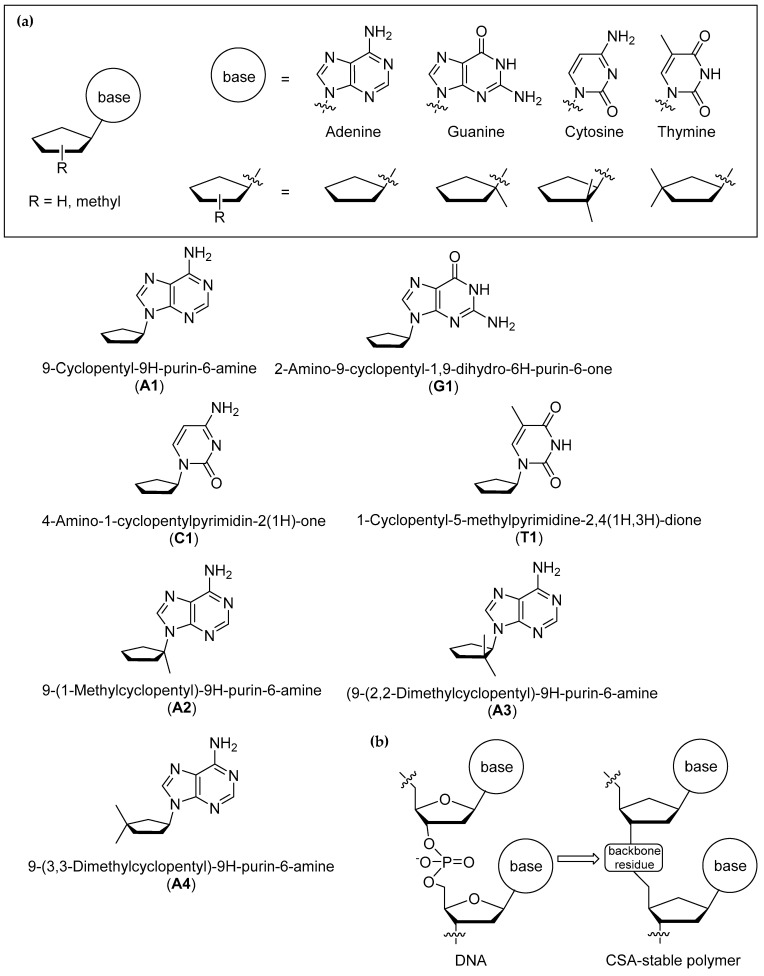
(**a**) Seven carbocyclic nucleosides studied in this work. (**b**) General schematic of the structure of the genetic-like polymer stable in concentrated sulfuric acid (CSA).

Here, we explore the cyclopentane motif as a candidate structural analog of the (deoxy)ribose linker in DNA and RNA (Figure 1). Cyclopentane nucleosides (and, more generally, carbocyclic nucleosides) have been studied as potential antiviral drugs for decades [31,32]. The cyclopentene nucleosides carbovir and its prodrug abacavir, as potent and selective anti-HIV agents, are prime examples of the antiviral activity of this family of compounds (reviewed in [33]).

To evaluate cyclopentane as a potential linker residue in genetic-like polymers that are stable in concentrated sulfuric acid, we synthesized seven cyclopentane nucleosides and tested their stability in 98% *w*/*w* sulfuric acid at room temperature (RT). We employed ^13^C and ^1^H Nuclear Magnetic Resonance Spectroscopy (NMR) to monitor the integrity of these molecules in 98% *w*/*w* sulfuric acid at RT over periods ranging from hours to weeks. We also describe detailed synthetic routes for all seven investigated compounds.

## 2. Results

We tested the stability of seven cyclopentyl nucleosides dissolved in D_2_SO_4_ (98 wt%, 2 wt% D_2_O). The ^1^H and ^13^C NMR spectra were measured after 1–2 h, 2 days, 7 days and 14 days (Table 1). The stability and reactivity of cyclopentyl nucleosides have not been previously tested in concentrated sulfuric acid. Here, we consider the compounds to be stable if we see no detectable reactivity and degradation of the tested compounds after incubation in 98% *w*/*w* sulfuric acid for up to 14 days at room temperature (RT). We first analyzed the compounds after 1 h of incubation in 98% *w*/*w* sulfuric acid and next repeated the NMR measurements after two and seven days, and finally after two weeks for an additional final stability test. By stable, we also mean the long-term (~2 weeks) stability of the heavy atom bonding topology in the tested compounds, i.e., the bonding of nonhydrogen atoms to each other, and not a rapid, reversible exchange of protons between the bases and the solvent. Such rapid exchange of hydrogen atoms is expected in sulfuric acid and is not a sign of the instability of the tested compounds [1,6,24,34,35].

We chose 98% *w*/*w* concentrated sulfuric acid for the stability experiments for two reasons. First, the concentration of sulfuric acid in the clouds of Venus varies with altitude, from 81% *w*/*w* acid in the top clouds, at 56.5–70 km above the surface, to 98% *w*/*w* acid in the lower cloud region (47.5–50.5 km) [36,37,38,39]. At 98% *w*/*w*, the amount of water is very low and sulfuric acid dominates the chemistry of the solvent, potentially entirely changing the expected chemical behavior of the investigated compounds, as compared with more diluted acid [27,28]. Second, the early experiments on the stability and reactivity of organic molecules, including nucleic acid bases, often focused on similarly high concentrations of acid [1,6,8,34,35].

We first tested the stability of four cyclopentane nucleosides (**A1**, **G1**, **C1**, **T1**), where the cyclopentane linker bonds directly to four different nucleic acid bases: adenine (**A1**), guanine (**G1**), thymine (**T1**) and cytosine (**C1**) (see Section 2.1). In Section 2.2, we explore how the position of the methyl group substituent in the cyclopentane ring influences the reactivity of the tested compound in 98% *w*/*w* sulfuric acid. In compound **A2,** we tested the reactivity of the cyclopentyl linker with two substitutions at Position 1 in the cyclopentane ring, i.e., the base and the methyl group bonded to the carbon C1 in the ring. In compound **A3,** we examined the reactivity of the cyclopentyl linker with two methyl groups bonded to the second (C2) position in the cyclopentane ring. Finally, in compound **A4**, we tested the reactivity of the cyclopentyl linker with two methyl groups bonded to the third (C3) position in the cyclopentane ring. The addition of the methyl groups at these specific positions in the cyclopentane ring aims to mimic the connection to other nucleosides in the backbone of the polymer.

### 2.1. Stability and Reactivity of Adenine, Guanine, Cytosine and Thymine Cyclopentane Nucleosides (***A1***, ***G1***, ***C1***, ***T1***) in 98% w/w Sulfuric Acid

**Figure 2:**
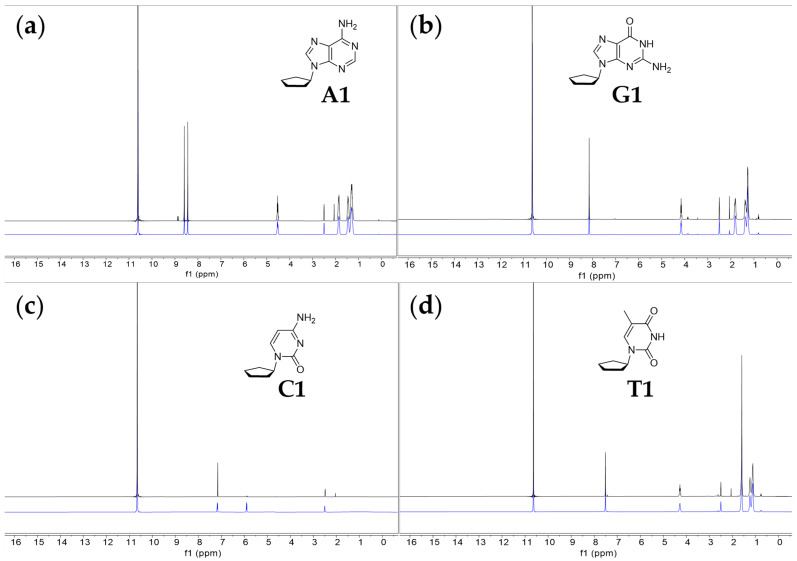
Comparison of the ^1^H NMR spectra of carbocyclic nucleosides in 98% D_2_SO_4_ and 2% D_2_O, by weight, with reference DMSO-*d*_6_ at room temperature, collected after 1–2 h (blue spectra) and and 2 weeks (black spectra) of incubation in acid. (**a**) ^1^H NMR spectra of compound **A1**; (**b**) ^1^H NMR spectra of compound **G1**; (**c**) ^1^H NMR spectra of compound **C1**; (**d**) ^1^H NMR spectra of compound **T1**. Compounds **G1** and **T1** remain stable in 98% *w*/*w* D_2_SO_4_ for at least two weeks at RT. Compound **A1** shows only very slight signs of degradation (~4%), while **C1** readily decomposes to soluble cytosine in concentrated sulfuric acid.

**Figure 3:**
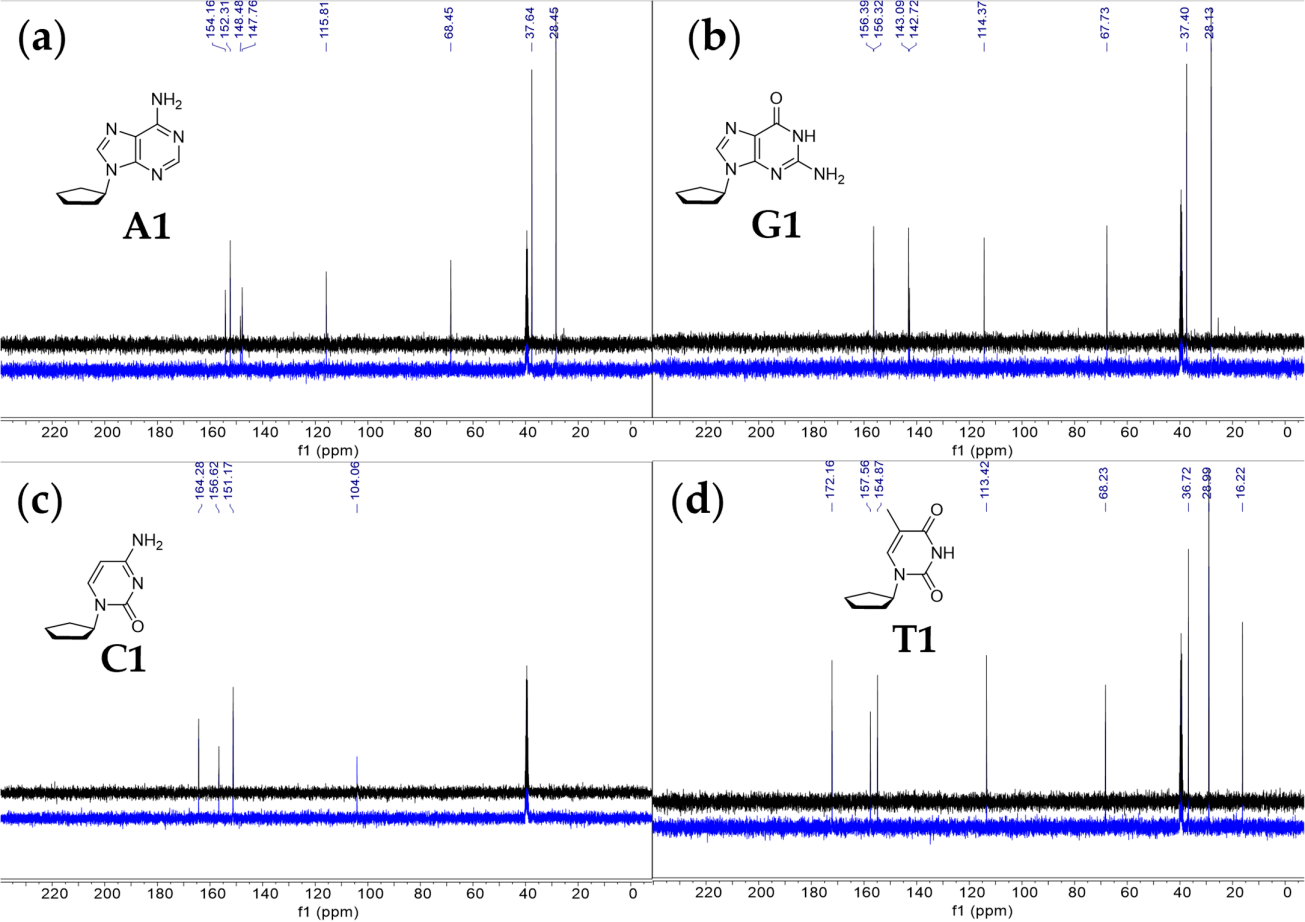
Comparison of ^13^C NMR spectra of carbocyclic nucleosides in 98% D_2_SO_4_ and 2% D_2_O, by weight, with reference DMSO-*d*_6_ at room temperature, collected after 1–2 h (blue spectra) and 2 weeks (black spectra) of incubation in acid. (**a**) ^13^C NMR spectra of compound **A1**; (**b**) ^13^C NMR spectra of compound **G1**; (**c**) ^13^C NMR spectra of compound **C1**; (**d**) ^13^C NMR spectra of compound **T1**. Compounds **G1** and **T1** remain stable in 98% *w*/*w* D_2_SO_4_ for at least two weeks at RT. Compound **A1** shows only very slight signs of degradation (~4%, Figure 2), while **C1** readily decomposes to soluble cytosine in concentrated sulfuric acid.

Out of the four tested cyclopentane nucleosides, three are soluble and stable in 98% *w*/*w* sulfuric acid (Figure 2 and Figure 3). The ^1^H and ^13^C NMR spectra of the compounds (**A1**, **G1**, **T1**) do not change significantly over the span of the two-week incubation in 98% *w*/*w* sulfuric acid. The cytosine cyclopentane nucleoside (**C1**) is highly reactive in 98% *w*/*w* sulfuric acid. The C–N bond connecting the base to the cyclopentane ring readily breaks in 98% *w*/*w* sulfuric acid, leaving behind acid-soluble cytosine. The cyclopentane itself is immiscible and insoluble in sulfuric acid. We note that over time, the ^1^H NMR signal corresponding to the H5 hydrogen (~5.8 ppm) in cytosine splits and broadens (Figure 2). The splitting and broadening of the H5 peak indicate an exchange of the H5 proton of the cytosine ring with the D_2_SO_4_ deuterium (i.e., H/D exchange) and is not a sign of an instability of the pyrimidine ring. Such an H/D exchange readily happens in acidic solutions [40]. Splitting and broadening, consistent with efficient H/D exchange and possible carbocation formation, is also visible in the ^13^C NMR spectrum of **C1**, where the C5 carbon signal of the cytosine nucleobase splits and broadens over time. We expect such splitting to happen, as aromatic systems often undergo efficient hydrogen exchange with the solvent, which could potentially lead to the formation of the stable carbocation [41,42,43]. Formation of the carbocation could also be consistent with the ^1^H NMR spectrum, where the H5 hydrogen peak also splits heavily and broadens. We have observed similar behavior of cytosine nucleic acid bases before, when they were incubated over long period of time in concentrated sulfuric acid [24,25].

We now turn to the detailed NMR assignment of the cyclopentane nucleosides **A1**, **G1** and **T1** that are stable in 98% *w*/*w* sulfuric acid, focusing on the spectra collected after 2 weeks of incubation in 98% *w*/*w* sulfuric acid at RT.

For compound **A1**, in the ^13^C NMR spectrum (Figure 3), we find five peaks that correspond to the five carbons in the adenine ring and three peaks corresponding to five atoms of the cyclopentane ring. Each of the five peaks corresponding to the adenine ring reside in the region of the NMR spectrum associated with aromatic compounds (peaks δ 154.2, 152.3, 148.4, 147.8 and 115.8). The ^13^C NMR spectrum matches the chemical shifts of adenine from our previous NMR studies in 98% *w/w* sulfuric acid [24,25]. We assign the three remaining carbon peaks to the aliphatic cyclopentane carbons. The peak at 68.5 ppm is significantly more deshielded than the other two peaks and corresponds to the cyclopentane carbon C1 attached to adenine’s imidazole ring nitrogen. The most upfield peak at 28.5 ppm corresponds to the two symmetric carbon atoms of the cyclopentane ring that are the furthest from the adenine ring. The peak at 37.6 ppm corresponds to the two symmetric carbon atoms of the cyclopentane ring, which experience a moderate deshielding effect and are bonded to the carbon atom that is directly connected to the adenine ring. The ^1^H NMR spectra are also consistent with compound **A1** being largely stable at RT for at least 2 weeks. We observe only very slight degradation (~4%) of the compound **A1**, as estimated from the ^1^H NMR spectrum. We also note that the chemical shift trends for the ^1^H and ^13^C NMR spectra agree with the values of ^13^C and ^1^H NMR collected in CDCl_3_ solvent (see Section 4.1 in Materials and Methods).

For compound **G1**, in the ^13^C NMR spectrum (Figure 3), we find five peaks that correspond to the five carbons in the guanine ring and three peaks corresponding to five atoms of cyclopentane ring. Each of the five peaks corresponding to the guanine ring reside in the region of the NMR spectrum associated with aromatic compounds (peaks δ 156.4, 156.3, 143.1, 142.7 and 114.4). The ^13^C NMR spectrum matches the chemical shifts of guanine from our previous NMR studies in 98% *w*/*w* sulfuric acid [24,25]. We assign the three remaining carbon peaks to the aliphatic cyclopentane carbons. The peak at 67.7 ppm is significantly more deshielded than the other two peaks and corresponds to the cyclopentane carbon attached to guanine’s imidazole ring nitrogen. The most upfield peak at 28.1 ppm corresponds to the two carbon atoms of the cyclopentane ring that are the furthest from the guanine ring. The peak at 37.4 ppm corresponds to the two symmetric carbon atoms of the cyclopentane ring, which experience a moderate deshielding effect and are bonded to the carbon atom that is directly connected to the guanine ring. The ^1^H NMR spectra are also consistent with compound **G1** being stable at RT for at least 2 weeks. We observe no degradation of the compound **G1**, as estimated from the ^1^H NMR spectrum. No new peaks emerge over the span of 2 weeks of incubation, confirming the overall stability of the compound **G1** in 98% *w*/*w* sulfuric acid. We also note that the chemical shift trends for the ^1^H and ^13^C NMR spectra agree with the values of ^13^C and ^1^H NMR collected in CDCl_3_ solvent (see Section 4.2 in Materials and Methods).

Finally, for compound **T1**, in the ^13^C NMR spectrum (Figure 3), we find four peaks that correspond to the four carbons in the thymine ring and three peaks corresponding to five carbon atoms of the cyclopentane ring. Each of the four peaks corresponding to the thymine ring reside in the region of the NMR spectrum associated with aromatic compounds (peaks δ 172.2, 157.6, 154.9 and 113.4). The ^13^C NMR spectrum generally matches the chemical shifts of thymine from our previous NMR studies in 98% *w*/*w* sulfuric acid [24,25]. We assign the three remaining carbon peaks to the aliphatic cyclopentane carbons. The peak at 68.2 ppm is significantly more deshielded than the other two peaks and corresponds to the cyclopentane carbon attached to thymine’s ring nitrogen. The most upfield peak of the three, at 29.0 ppm, corresponds to the two carbon atoms of the cyclopentane ring that are the furthest from the thymine ring. The peak at 36.7 ppm corresponds to the two symmetric carbon atoms of the cyclopentane ring, which experience a moderate deshielding effect and are bonded to the carbon atom directly connected to the thymine ring. Finally, we assign the peak at 16.2 ppm to the methyl group carbon of the thymine nucleobase. The ^1^H NMR spectra are also consistent with compound **T1** being stable at RT for at least 2 weeks. We observe no degradation of the compound **T1**, as estimated from the ^1^H NMR spectrum. No new peaks emerge over the span of 2 weeks of incubation, confirming the overall stability of the compound **T1** in 98% *w*/*w* sulfuric acid. We also note that the chemical shift trends for ^1^H and ^13^C NMR spectra agree with the values of ^13^C and ^1^H NMR collected in CDCl_3_ solvent (see Section 4.4 in Materials and Methods).

### 2.2. Stability and Reactivity of Modified Adenine Cyclopentane Nucleosides (***A2***, ***A3***, ***A4***) in 98% w/w Sulfuric Acid

**Figure 4:**
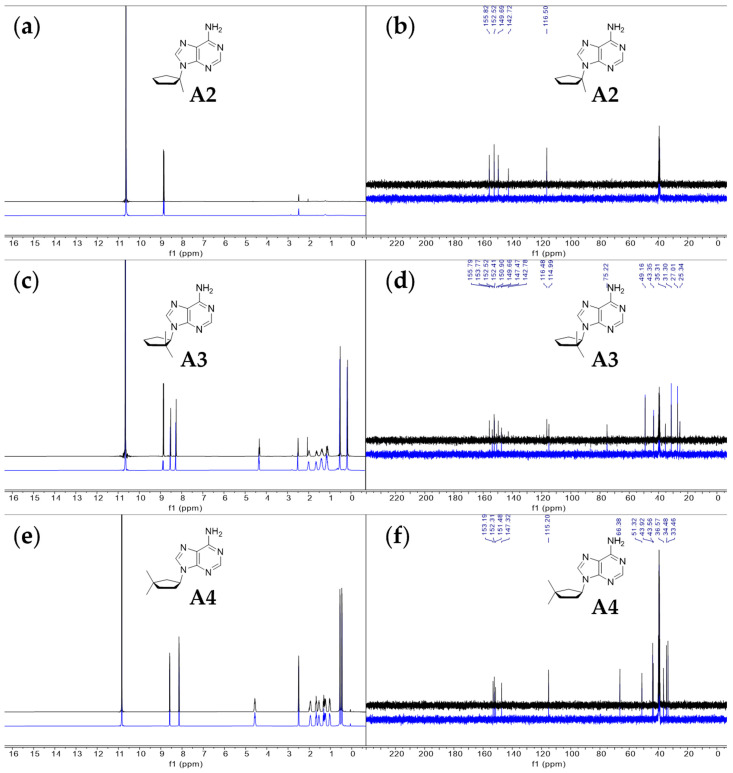
Comparison of the NMR spectra of the modified carbocyclic nucleosides (**A2**, **A3**, **A4**) in 98% D_2_SO_4_ and 2% D_2_O, by weight, with reference DMSO-*d*_6_ at room temperature, collected after 1–2 h (blue spectra) and 2 weeks (black spectra) of incubation in acid. (**a**) ^1^H NMR spectra of compound **A2**; (**b**) ^13^C NMR spectra of compound **A2**; (**c**) ^1^H NMR spectra of compound **A3**; (**d**) ^13^C NMR spectra of compound **A3**; (**e**) ^1^H NMR spectra of compound **A4**; (**f**) ^13^C NMR spectra of compound **A4**. Out of the three tested compounds, only compound **A4** remains stable in 98% *w*/*w* D_2_SO_4_ for at least two weeks at RT.

Having established that, overall, compounds **A1**, **G1** and **T1** remain stable in 98% *w*/*w* sulfuric acid, we investigated how the cyclopentane ring substituents affect the stability of the nucleoside structure. We modified compound **A1** to include additional methyl groups at various positions in the cyclopentane ring (compounds **A2**, **A3** and **A4** in Figure 1). We show that compounds **A2** and **A3** are unstable in concentrated sulfuric acid, leading to the release of the soluble adenine nucleobase (Figure 4). Cleavage of the branched-carbocycle adenine connection in compound **A2** is expected, a tertiary carbocation forms more readily, is more stable, and acts as a better leaving group than a secondary carbocation that forms in the cyclopentane variant **A1**. Carbocations form as a result of the exchange of H between tertiary carbon and the sulfuric acid solvent. Such exchange leads to the formation of the reactive meta-stable species with positively charged carbon atoms (carbocations). The carbocation formation is only efficient in aromatic compounds (see compound **C1** above) or alkyl groups with tertiary carbon groups (in **A2**) and not in CH_2_ (in **A1**) or in methyl (CH_3_) groups themselves [42]. Compound **A3** degrades slowly over time, and after 14 days, 48% of **A3**, as estimated by ^1^H NMR, converts to a soluble nucleobase adenine (Figure 4).

Out of the three tested analogs of compound **A1**, only the cyclopentane nucleoside **A4** is stable in 98% *w*/*w* sulfuric acid. For compound **A4**, in the ^13^C NMR spectrum (Figure 4), we find five peaks that correspond to the five carbons in the adenine ring and seven peaks corresponding to five atoms of cyclopentane ring and two methyl group substituents. Each of the five peaks corresponding to the adenine ring reside in the region of the NMR spectrum associated with aromatic compounds (peaks δ 153.2, 152.3, 151.5, 147.3 and 115.2). The ^13^C NMR spectrum matches the chemical shifts of adenine from our previous NMR studies in 98% *w*/*w* sulfuric acid [24,25]. The remaining seven carbon peaks (peaks δ 66.4, 51.3, 43.9, 43.6, 36.6, 34.5 and 33.5) correspond to the 3,3-dimethylcyclopentyl ring, with the two most upfield peaks (34.5 ppm and 33.5 ppm) corresponding to the two methyl group substituents. The ^1^H NMR spectra are also consistent with compound **A4** being stable at RT for at least 2 weeks. We observe no degradation of the compound **A4**, as estimated from the ^1^H NMR spectrum. No new peaks emerge over the span of 2 weeks of incubation, confirming the overall stability of the compound **A4** in 98% *w*/*w* sulfuric acid. We also note that the chemical shift trends for ^1^H and ^13^C NMR spectra agree with the values of ^13^C and ^1^H NMR collected in CDCl_3_ solvent (see Section 4.7 in Materials and Methods).

## 3. Discussion

Our work is a part of a larger effort towards the design and synthesis of a structural analog of DNA capable of forming a double-helix-like structure in concentrated sulfuric acid. DNA itself rapidly degrades in this aggressive solvent due to the solvolysis of deoxyribose residues and the phosphodiester backbone of the polymer. Therefore, the stability of a new linker (substituting the reactive deoxyribose) and backbone (instead of the phosphate ester) residues need to be designed and tested.

Reactivity tests of the cyclopentane linker, as a structural analog of deoxyribose, are a first step towards this goal. We note that replacing the ribose sugar with a cyclopentane scaffold may reduce the intrinsic chemical and stereochemical functionality available to the backbone itself, even though additional functional groups could, in principle, be introduced elsewhere in the polymer.

Our results show that the reactivity of cyclopentane nucleosides in 98% *w*/*w* sulfuric acid varies significantly with respect to the nucleobase attached to the cyclopentane linker (Table 1). The differences in reactivity amongst nucleosides **A1**, **G1**, **C1** and **T1** in 98% *w*/*w* sulfuric acid at RT pose a potential problem for the design of the polymer with a cyclopentane linker. If the stability of all four nucleosides is not the same, then such linker–base connections could lead to different sequence-dependent reactivity of the whole polymer. The use of cyclopentane in the design of such a polymer might therefore require substituting reactive canonical bases, like cytosine, with other less reactive counterparts (see the discussion below).

**Figure 5:**
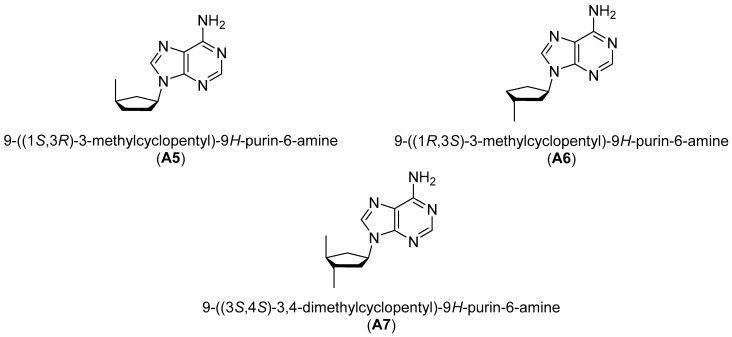
Compounds with methyl substituents at different positions, as relevant to the synthesis and stability experiments of the genetic-like polymer in concentrated sulfuric acid. If the separation of different diastereoisomers of compounds **A5**, **A6** and **A7** proves to be possible, then the sulfuric acid reactivity of individual diastereoisomers will be attempted. Otherwise, a stability study will be performed on diastereoisomeric mixtures.

We also explored the differences in the reactivity of the cyclopentane with respect to the position of the attachment of the linker ring to the backbone of the polymer. In compounds **A2**, **A3** and **A4**, the position of the additional methyl groups corresponds to the potential attachment points to the rest of the polymer (Figure 1). Our reactivity experiments show that the arrangement of the methyl groups in the cyclopentane ring highly influences the stability of the entire nucleoside in 98% *w*/*w* sulfuric acid. In other words, the stability of the cyclopentane nucleosides **A2**, **A3** and **A4** differs with respect to the position of the additional methyl groups attached to the ring. This observation is important, as the place of attachment of the backbone residue to the cyclopentane linker could influence the stability of the entire polymer. Our reactivity experiments show that the attachment of the backbone residues at the C1 or C2 positions in the cyclopentane ring could result in the instability of the entire polymer, as exemplified by the overall instability of compounds **A2** and **A3** (Table 1). On the other hand, the fact that compound **A4** is stable suggests that the backbone residue connected at position C3 in the cyclopentane linker might lead to a stable polymer structure. In follow-up studies, we plan to explore the reactivity of other substituents at other positions (positions C3 and C4 in compounds **A5**, **A6** and **A7** in Figure 5) to fully cover the structural chemical space of possible connections to the stable backbone of the polymer.

More work is needed for a full assessment of the viability of cyclopentanes as potential linker residues that are stable in concentrated sulfuric acid. Specifically, stability assessments of selected modified cyclopentanes at higher temperatures, spanning the 80–100 °C temperature range of the Venusian cloud deck, are needed. For example, we previously demonstrated that Peptide Nucleic Acid (PNA) could be a promising candidate for a genetic polymer that is stable in concentrated sulfuric acid [44]. PNA, however, while stable at room temperature in 98% *w*/*w* sulfuric acid, decomposes above 50 °C. PNA’s solvolysis at high temperatures proceeds by cleavage of a single tertiary amide bond in an acetyl group linker that connects the nucleic acid base to the backbone of the polymer. The solvolysis yields two distinct products that appear to be stable to further degradation in 98% *w*/*w* sulfuric acid [44]. The thermal instability of PNA’s acetyl group linker prompts us to seek other structural analogs to DNA in parallel, in an effort to identify at least several examples of chemical motifs that could serve as stable backbone and linker residues of a genetic-like polymer in concentrated sulfuric acid. The thermal stability of modified cyclopentane nucleosides at temperatures above 50 °C should be part of these future efforts.

The next stages in the design of the genetic-like polymer also include finding an alternative to the reactive DNA phosphate ester backbone. We plan to explore phosphinic acid and amine backbone polymers. A phosphinic acid-based backbone residue exchanges the –O–P–O– phosphate ester bonds for an acid stable –C–P–C– phosphinic backbone. Such a phosphinic backbone, however, would likely lose its permanent repeating negative charge due to efficient protonation in concentrated sulfuric acid. An amine might therefore be an especially promising candidate for an alternative backbone. In concentrated sulfuric acid, amines are fully and stably protonated. Recall that a permanent repeating charge, no matter if it is negative (as phosphates in DNA in water) or positive (as in amines in concentrated sulfuric acid), is likely a universal requirement for any genetic polymer of life, regardless of its biochemical makeup [45,46,47].

We are aware, however, that even with a stable alternative linker and backbone, such as that of PNA [44], or modified cyclopentane, it is likely that the protonation and tautomeric forms of the adenine, thymine, guanine and cytosine bases will be different in concentrated sulfuric acid than in water. As a result, classical Watson–Crick base pairing may not be possible between complementary strands containing canonical A, T, C, G bases, and alternative bases, that do not rely on hydrogen bonding to pair, may be necessary for life to survive in this solvent [48]. Such alternative nucleobases that pair exclusively via hydrophobic and van der Waals interactions have been routinely explored in synthetic biology (e.g., [49,50,51,52,53,54]).

In summary, we find that cyclopentane is a promising structural motif for the linker residue of a genetic-like polymer that is stable in 98% *w*/*w* sulfuric acid at room temperature. Future stability experiments should test the thermal stability of the cyclopentane linker, extend the cyclopentane scaffold to other modified variants to test additional substitution patterns (**A5**–**A7**) and culminate in an effort to pair stable cyclopentane linkers with alternative backbone chemistries. Together, our experiments aim to identify at least one robust candidate for a genetic-like polymer that could potentially persist in the sulfuric acid clouds of Venus in order to motivate space missions to search in situ in the Venusian atmosphere for signs of life or life itself.

## 4. Materials and Methods

We confirmed the structural integrity of the synthesized compounds with Liquid Chromatography–Mass Spectrometry (LCMS) and NMR spectroscopy. We used LCMS as a supporting technique to NMR to identify the intermediate as well as the final products of cyclopentane nucleoside synthesis. We used MNova software, version: 14.3.0-30573 (Mestrelab Research, Barcelona, Spain) to process and analyze the NMR data [55]. The original data for all LCMS and NMR spectra are available on Zenodo at https://zenodo.org/records/18441685.

### 4.1. Synthesis of 9-Cyclopentyl-9H-purin-6-amine (***A1***)

Compound **A1** was synthesized in two steps starting with a Mitsunobu reaction between Alcohol **1** and Compound **2**, followed by amination with ammonia. Compound **A1** was obtained with 76% yield over two steps.

**Scheme 1:**
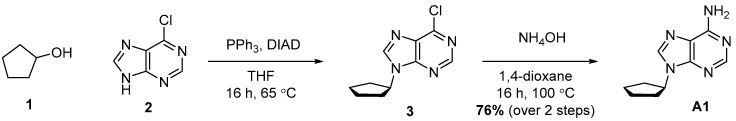
Synthesis of compound **A1**.

Cyclopentanol (500 mg, 527 μL, 1 equiv., 5.81 mmol), 6-chloro-9H-purine (1.08 g, 1.2 equiv., 6.97 mmol) and triphenylphosphine (1.83 g, 1.2 equiv., 6.97 mmol) were dissolved in THF (58 mL). DIAD (1.35 mL, 1.2 equiv., 6.97 mmol) was added and the mixture was refluxed for 16 h. The mixture was concentrated in vacuo and then purified by column chromatography (40 g silica, 0–100% EtOAc/heptane gradient) to obtain the slightly impure Intermediate **3** (1.27 g). Intermediate **3** was dissolved in 1,4-dioxane (6.0 mL) and transferred to a pressure tube. Ammonia (6.0 mL, 25 wt%, 12 equiv., 69 mmol) was added and the reaction mixture was heated to 100 °C for 16 h. The mixture was partitioned between brine and EtOAc. We extracted the aqueous layer once with EtOAc again. Combined organic layers were dried over Na_2_SO_4_, filtered and concentrated in vacuo. The crude extract was purified by column chromatography (25 g silica, 0–4% MeOH/DCM gradient), and Product **A1** (900 mg, 4.43 mmol, 76% yield over 2 steps) was obtained as a white solid (Scheme 1). **^1^H NMR (400 MHz, CDCl_3_)** had peaks at δ 8.36 (s, 1H), 7.85 (s, 1H), 5.85 (s, 2H), 4.92 (p, *J* = 7.3 Hz, 1H), 2.36–2.20 (m, 2H) and 2.07–1.71 (m, 6H). **^13^C NMR (101 MHz, CDCl_3_)** had peaks at δ 155.5, 152.7, 150.3, 138.8, 120.1, 56.1, 32.9 and 24.0. Compound **A1** was dissolved in 98 wt% D_2_SO_4_ and DMSO-*d_6_* (9/1 *v*/*v*), and NMR spectra were measured: **^1^H NMR (400 MHz, D_2_SO_4_)**, δ 8.59 (s, 1H), 8.44 (d, *J* = 1.2 Hz, 1H), 4.58–4.46 (m, 1H), 1.92–1.79 (m, 2H), 1.52–1.39 (m, 2H) and 1.38–1.21 (m, 4H); **^13^C NMR (101 MHz, D_2_SO_4_)**, δ 154.2, 152.3, 148.4, 147.8, 115.8, 68.5, 37.6 and 28.5.

### 4.2. Synthesis of 2-Amino-9-cyclopentyl-1,9-dihydro-6H-purin-6-one (***G1***)

Compound **G1** was synthesized in two steps, starting with a Mitsunobu reaction between Alcohol **1** and Compound **4**, followed by hydrolysis with aqueous TFA. Compound **G1** was obtained with 41% yield over two steps.

**Scheme 2:**
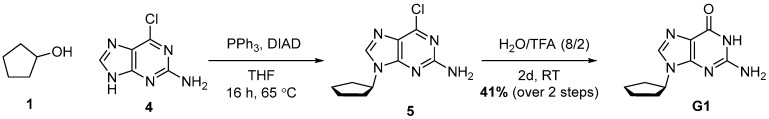
Synthesis of compound **G1**.

To a mixture of cyclopentanol (1.33 g, 1.40 mL, 1 equiv., 15.4 mmol), 6-chloro-9H-purin-2-amine (3.93 g, 1.5 equiv., 23.2 mmol), triphenylphosphine (6.08 g, 1.5 equiv., 23.2 mmol) and THF (58 mL), DIAD (4.68 g, 4.50 mL, 1.5 equiv., 23.2 mmol) was added at RT. The mixture was then heated to reflux for 16 h and concentrated in vacuo. The crude extract was purified by column chromatography (120 g silica, 0–4% MeOH/DCM gradient) to obtain Intermediate **5** (4.1 g) with triphenylphosphine oxide as an impurity. Impure Intermediate **5** was dissolved in TFA/H_2_O (8/2 *v*/*v*, 90 mL) and stirred at RT for 2 days. The mixture was concentrated in vacuo and co-evaporated with toluene. The residue was suspended in Et_2_O by sonication, filtered over a glass filter, washed with Et_2_O and then dried in vacuo. Product **G1** (1,41 g, 41% yield over 2 steps) was obtained as a white solid (Scheme 2). **^1^H NMR (400 MHz, DMSO)** had peaks at δ 10.78 (s, 1H), 7.74 (s, 1H), 6.48 (s, 2H), 4.59 (p, *J* = 7.4 Hz, 1H), 2.12–1.96 (m, 2H), 1.94–1.74 (m, 4H) and 1.74–1.56 (m, 2H). **^13^C NMR (101 MHz, DMSO)** had peaks at δ 157.5, 153.7, 151.1, 135.3, 116.9, 54.7, 32.0 and 23.4. Compound **G1** was dissolved in 98 wt% D_2_SO_4_ and DMSO-*d_6_* (9/1 *v*/*v*), and the NMR spectra were measured: **^1^H NMR (400 MHz, D_2_SO_4_)**, δ 8.14 (s, 1H), 4.26–4.07 (m, 1H), 1.91–1.75 (m, 2H) and 1.33 (d, *J* = 44.5 Hz, 6H); **^13^C NMR (101 MHz, D_2_SO_4_)**, δ 156.4, 156.3, 143.1, 142.7, 114.4, 67.7, 37.4 and 28.1.

### 4.3. Synthesis of 4-Amino-1-cyclopentylpyrimidin-2(1H)-one (***C1***)

Compound **C1** was synthesized in two steps, starting with a Mitsunobu reaction between Alcohol **1** and Compound **6**, followed by deacylation with ammonia. Compound **C1** was obtained with 33% yield over two steps.

**Scheme 3:**
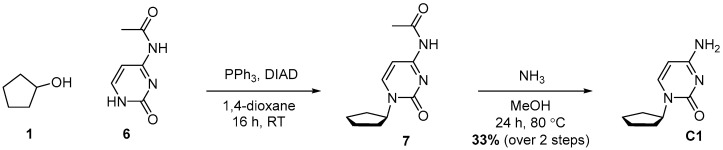
Synthesis of compound **C1**.

To a mixture of cyclopentanol (609 mg, 642 μL, 1 equiv., 7.07 mmol), N-(2-oxo-1,2-dihydropyrimidin-4-yl)acetamide (1.19 g, 1.1 equiv., 7.78 mmol), triphenylphosphine (2.23 g, 1.2 equiv., 8.48 mmol) and 1,4-dioxane (70 mL), DIAD (1.65 mL, 1.2 equiv., 8.48 mmol) was added dropwise over 5 min. The mixture was then sonicated for 1 min and stirred at RT for 16 h. The mixture was concentrated in vacuo, and the crude extract was purified by column chromatography (120 g silica, 0–40% EtOAc/heptane gradient) to obtain Intermediate **7**. Compound **7** was dissolved in MeOH (6.0 mL) and transferred to a pressure tube, and NH_3_ in MeOH (4.7 g, 6.0 mL, 7.0 molar, 5.9 equiv., 42 mmol) was added. The mixture was heated to 80 °C for 24 h and then concentrated in vacuo. The crude mixture was purified by column chromatography (40 g silica, 20–40% EtOAc/heptane gradient) to obtain Product **C1** (417 mg, 33% yield over 2 steps) as a white solid (Scheme 3). **^1^H NMR (400 MHz, CDCl_3_)** had peaks at δ 8.00 (d, *J* = 5.7 Hz, 1H), 6.06 (d, *J* = 5.7 Hz, 1H), 5.34 (tt, *J* = 6.1, 3.0 Hz, 1H), 4.86 (s, 2H), 2.00–1.71 (m, 6H) and 1.69–1.51 (m, 2H). **^13^C NMR (101 MHz, CDCl_3_)** had peaks at δ 165.2, 164.7, 157.5, 99.1, 79.2, 33.0 and 23.9. Compound **C1** was dissolved in 98 wt% D_2_SO_4_ and DMSO-*d_6_* (9/1 *v*/*v*), and the NMR spectra were measured. Only cytosine was visible.

### 4.4. Synthesis of 1-Cyclopentyl-5-methylpyrimidine-2,4(1H,3H)-dione (***T1***)

Compound **T1** was synthesized in one step via a Mitsunobu reaction between Alcohol **1** and Compound **8**. Compound **T1** was obtained with 15% yield after multiple purifications.

**Scheme 4:**
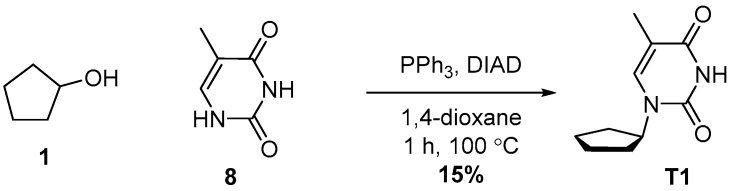
Synthesis of compound **T1**.

Cyclopentanol (1.4 g, 2 mL, 1.0 equiv., 16 mmol), 5-methylpyrimidine-2,4(1H,3H)-dione (2.0 g, 1 equiv., 16 mmol) and triphenylphosphine (6.2 g, 1.5 equiv., 24 mmol) were dissolved in 1,4-dioxane (40 mL). The mixture was then heated to reflux, after which DIAD (4.8 g, 4.6 mL, 1.5 equiv., 24 mmol) was added in dropwise fashion. After addition, refluxing was continued for another hour, after which LCMS showed complete conversion to the desired product. The mixture was concentrated in vacuo, and the crude extract was subjected to purification by silica column chromatography to produce 3.5 g of the desired material with low purity. Following this, reverse phase chromatography was used to obtain Product **T1** (465 mg, 2.39 mmol, 15%) as a white solid (Scheme 4). **^1^H NMR (400 MHz, CDCl_3_)** had peaks at δ 8.66 (s, 1H), 7.02 (d, *J* = 1.1 Hz, 1H), 4.91 (p, *J* = 8.2 Hz, 1H), 2.19–2.01 (m, 2H), 1.94 (d, *J* = 1.2 Hz, 3H) and 1.88–1.53 (m, 6H). **^13^C NMR (101 MHz, CDCl_3_)** had peaks at δ 163.7, 151.2, 136.9, 111.0, 56.5, 31.4, 24.3 and 12.8. Compound **T1** was dissolved in 98 wt% D_2_SO_4_ and DMSO-*d_6_* (9/1 *v*/*v*), and the NMR spectra were measured: **^1^H NMR (400 MHz, D_2_SO_4_)**, δ 7.51 (s, 1H), 4.35–4.19 (m, 1H), 1.59 (s, 5H) and 1.18 (d, *J* = 47.8 Hz, 6H); **^13^C NMR (101 MHz, D_2_SO_4_)**, δ 172.2, 157.6, 154.9, 113.4, 68.2, 36.7, 29.0 and 16.2.

### 4.5. Synthesis of 9-(1-Methylcyclopentyl)-9H-purin-6-amine (***A2***)

The synthesis of 1-methyl substituted **A1** started with the synthesis of tertiary amine **11**, because the substitution on tertiary alcohol **9** is not expected to work. Amine **11** was prepared from Alcohol **9** in two steps via a Ritter reaction with 48% yield. Next, Amine **11** was converted to **A2** in three steps with 22% yield.

**Scheme 5:**
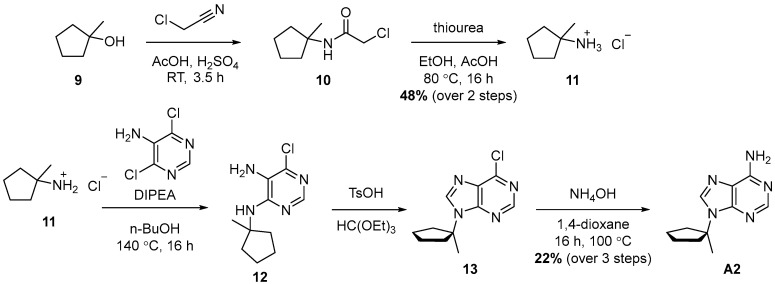
Synthesis of compound **A2**.

To a mixture of 1-methylcyclopentan-1-ol (2.0 g, 1 equiv., 20 mmol), chloroacetonitrile (2.3 g, 1.9 mL, 1.5 equiv., 30 mmol) and AcOH (2.9 g, 2.8 mL, 2.4 equiv., 49 mmol) at 0 °C, H_2_SO_4_ was added (5.2 g, 2.8 mL, 2.6 equiv., 53 mmol) dropwise over 2–3 min. The mixture was stirred for 3.5 h at RT, whereupon ice-cold water (50 mL) and tBME (50 mL) were added. The organic layer was washed twice with sat. NaHCO_3_, with brine, dried over Na_2_SO_4_, filtered and concentrated in vacuo. The resulting crude mixture of compound **10** (3.1 g), thiourea (1.5 g, 1 equiv., 20 mmol), EtOH (30 mL) and AcOH (6.0 mL) were heated to 80 °C for 16 h. The mixture was allowed to cool to RT, and water (60 mL) was added. The mixture was filtered, washed with water and then basified to pH 10 with NaOH (30 wt%). The aqueous layer was extracted with tBME (3x), and the combined organic layers were washed with brine, dried over Na_2_SO_4_ and filtered. The filtrate was acidified by adding HCl (10 mL, 4 M in dioxane) and then concentrated in vacuo. The green solids were triturated with Et_2_O, dissolved in a minimal amount of MeOH and precipitated by adding it to excess Et_2_O. The resulting precipitate was filtered, washed with Et_2_O and with pentane, and dried in vacuo. Product **11** (1.29 g, 48% yield over 2 steps) was obtained as a white solid (Scheme 5). **^1^H NMR (400 MHz, MeOD)** had peaks at δ 1.88–1.73 (m, 8H) and 1.41 (s, 3H).

A mixture of 1-methylcyclopentan-1-amine hydrochloride (**11**) (1.29 g, 1 equiv., 9.51 mmol), 4,6-dichloropyrimidin-5-amine (2.34 g, 1.5 equiv., 14.3 mmol), DIPEA (4.92 g, 6.63 mL, 4 equiv., 38.0 mmol) and n-butanol (25 mL) was heated to 140 °C (external) in a pressure tube for 16 h. The mixture was concentrated in vacuo, dissolved in EtOAc and washed with HCl (50 mL, 0.2 M), with sat. NaHCO_3_ and then with brine. The organic layer was dried over Na_2_SO_4_, filtered and concentrated in vacuo. The residue was purified by column chromatography (80 g silica, 0–30% EtOAc/heptane gradient) to obtain Intermediate **12** (650 mg, 2.87 mmol, 30%). To Compound **12** (650 mg) triethyl orthoformate (8.3 mL, 50 mmol) and p-toluenesulfonic acid monohydrate (38 mg, 0.20 mmol) were added, and the mixture was stirred at RT for 24 h. The mixture was concentrated in vacuo and to the resulting orange solid (**13**) ammonia (3.0 mL, 25 wt%, 39 mmol) and 1,4-dioxane (3 mL) were added. This mixture was heated in a pressure tube at 100 °C for 16 h. The reaction mixture was partitioned between EtOAc (25 mL) and brine (25 mL), and the aqueous layer was extracted with EtOAc. The combined organic layers were dried over Na_2_SO_4_, filtered and concentrated in vacuo. The residue was purified by column chromatography (25 g silica, 0–5% MeOH/DCM gradient) to obtain Product **A2** (458 mg, 2.11 mmol, 22% yield over 3 steps) as a white solid (Scheme 5). **^1^H NMR (400 MHz, CDCl_3_)** had peaks at δ 8.35 (s, 1H), 7.86 (s, 1H), 5.81 (s, 2H), 2.49 (dt, *J* = 11.6, 5.5 Hz, 2H), 2.24–2.08 (m, 2H), 1.82 (dq, *J* = 6.5, 3.2 Hz, 4H) and 1.69 (s, 3H). **^13^C NMR (101 MHz, CDCl_3_)** had peaks at δ 155.6, 152.1, 150.4, 139.0, 121.1, 66.8, 38.7, 26.4 and 22.8. Compound **A2** was dissolved in 98 wt% D_2_SO_4_ and DMSO-*d_6_* (9/1 *v*/*v*), and the NMR spectra were measured. Only adenine was visible.

### 4.6. Synthesis of (9-(2,2-Dimethylcyclopentyl)-9H-purin-6-amine (***A3***)

Compound **A3** was synthesized in two steps, starting with a Mitsunobu reaction between Alcohol **14** and Compound **2**, followed by amination with ammonia. Compound **A3** was obtained with 7% yield over two steps. The synthesis of **A3** was low-yielding because the first Mitsunobu step towards **15** is troublesome due to the steric hindrance of Alcohol **14**.

**Scheme 6:**
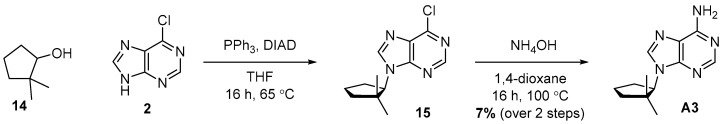
Synthesis of compound **A3**.

To a mixture of 2,2-dimethylcyclopentan-1-ol (451 mg, 1 equiv., 3.95 mmol), 6-chloro-9H-purine (1.22 g, 2 equiv., 7.90 mmol), triphenylphosphine (2.07 g, 2 equiv., 7.90 mmol) and THF (45 mL), DIAD (1.54 mL, 2 equiv., 7.90 mmol) was added at RT. The mixture was heated to 65 °C for 16 h and then concentrated in vacuo. The crude mixture was purified by column chromatography (80 g silica, 0–70% EtOAc/heptane gradient) to obtain impure Intermediate **15** (755 mg). This material was dissolved in 1,4-dioxane (4.0 mL) and transferred to a pressure tube, then ammonia (4.0 mL, 25 wt%, 12 equiv., 46 mmol) was added and the resulting mixture was heated to 100 °C for 16 h. The reaction mixture was partitioned between EtOAc and brine, and the aqueous layer was extracted with EtOAc. The combined organic layers were dried over Na_2_SO_4_, filtered and concentrated in vacuo. The residue was purified by column chromatography (25 g silica, 0–4% MeOH/DCM gradient) to obtain slightly impure Product **A3** (64 mg, 0.28 mmol, 7% yield over 2 steps) as a white solid (Scheme 6). **^1^H NMR (400 MHz, CDCl_3_)** had peaks at δ 8.36 (s, 1H), 7.84 (s, 1H), 5.87 (s, 2H), 4.71 (t, *J* = 8.1 Hz, 1H), 2.44–2.18 (m, 2H), 2.04–1.81 (m, 2H), 1.74–1.65 (m, 2H), 1.11 (s, 3H) and 0.75 (s, 3H). **^13^C NMR (101 MHz, CDCl_3_)** had peaks at δ 155.6, 152.8, 151.0, 139.6, 119.6, 63.8, 43.2, 39.2, 30.1, 27.7, 23.0 and 20.9. Compound **A3** was dissolved in 98 wt% D_2_SO_4_ and DMSO-*d_6_* (9/1 *v*/*v*) and the NMR spectra were measured: **^1^H NMR (400 MHz, D_2_SO_4_)**, δ 8.52 (s, 1H), 8.26 (s, 1H), 4.33 (t, *J* = 7.3 Hz, 1H), 2.05–1.91 (m, 1H), 1.70–1.56 (m, 1H), 1.47–1.29 (m, 2H), 1.21–1.03 (m, 2H), 0.51 (s, 3H) and 0.16 (s, 3H); **^13^C NMR (101 MHz, D_2_SO_4_)** δ 153.8, 152.4, 150.7, 147.5, 115.0, 75.3, 49.2, 43.3, 35.3, 31.3, 27.0 and 25.3. Note that **A3** degraded slowly over time to adenine in 98% *w*/*w* D_2_SO_4_.

### 4.7. Synthesis of 9-(3,3-Dimethylcyclopentyl)-9H-purin-6-amine (***A4***)

Synthesis of **A4** started with sodium borohydride reduction of Ketone **16** to Alcohol **17**. Alcohol **17** was obtained in 77% yield and then substituted with **2** under Mitsunobu conditions towards **18**. Treatment of **18** with ammonia gave **A4** with 53% yield over two steps.

To a mixture of 3,3-dimethylcyclopentan-1-one (1.95 g, 1 equiv., 17.4 mmol) in methanol (60 mL) at 0 °C, NaBH_4_ was added (789 mg, 1.2 equiv., 20.9 mmol) in small batches over 1 min. The mixture was stirred at RT for 3 h and then partitioned between water (75 mL) and Et_2_O (75 mL). The organic layer was washed with brine, dried over Na_2_SO_4_, filtered and concentrated in vacuo. The crude mixture was purified by column chromatography (40 g silica, 0–4% MeOH/DCM gradient) to obtain Product **17** (1.53 g, 77%) as a transparent oil. **^1^H NMR (400 MHz, CDCl_3_)** had peaks at δ 4.38 (tdd, *J* = 9.1, 4.5, 3.2 Hz, 1H), 2.06–1.94 (m, 1H), 1.79 (dd, *J* = 13.4, 6.8 Hz, 1H), 1.69–1.59 (m, 2H), 1.50 (s, 1H), 1.44–1.34 (m, 2H), 1.11 (s, 3H) and 0.96 (s, 3H).

**Scheme 7:**
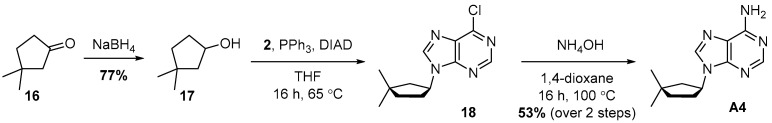
Synthesis of compound **A4**.

To a mixture of 3,3-dimethylcyclopentan-1-ol (**17**) (654 mg, 1 equiv., 5.73 mmol), 6-chloro-9H-purine (1.77 g, 2 equiv., 11.5 mmol), triphenylphosphine (3.00 g, 2 equiv., 11.5 mmol) and THF (40 mL), DIAD was added (2.23 mL, 2 equiv., 11.5 mmol) at RT. The mixture was heated to 65 °C for 16 h and then concentrated in vacuo. The crude extract was purified by column chromatography (120 g silica, 0–70% EtOAc/heptane gradient) to obtain impure Intermediate **18** (2.3 g). This material was dissolved in 1,4-dioxane (11.5 mL) and transferred to a pressure tube, then ammonia was added (11.5 mL, 25 wt%, 133 mmol) and then the resulting mixture was heated to 100 °C for 16 h. The reaction mixture was partitioned between EtOAc and brine, and the aqueous layer was extracted with EtOAc. The combined organic layers were dried over Na_2_SO_4_, filtered and concentrated in vacuo. The residue was purified by column chromatography (40 g silica, 0–4% MeOH/DCM gradient) to obtain Product **A4** (740 mg, 3.20 mmol, 56% yield over 2 steps) as a white solid (Scheme 7). **^1^H NMR (400 MHz, CDCl_3_)** had peaks at δ 8.36 (s, 1H), 7.89 (s, 1H), 5.83 (s, 2H), 5.02 (dq, *J* = 9.7, 8.1 Hz, 1H), 2.43 (dtd, *J* = 13.9, 7.9, 6.3 Hz, 1H), 2.20–2.12 (m, 1H), 2.11–2.02 (m, 1H), 1.92–1.75 (m, 2H), 1.63 (dtd, *J* = 13.0, 7.7, 0.8 Hz, 1H), 1.20 (s, 3H) and 1.12 (s, 3H). **^13^C NMR (101 MHz, CDCl_3_)** had peaks at δ 155.4, 152.5, 150.2, 138.7, 120.0, 55.2, 47.4, 39.2, 38.3, 32.4, 30.2 and 29.2. Compound **A4** was dissolved in 98 wt% D_2_SO_4_ and DMSO-*d_6_* (9/1 *v*/*v*), and the NMR spectra were measured: **^1^H NMR (400 MHz, D_2_SO_4_)**, δ 8.58 (d, *J* = 4.0 Hz, 1H), 8.14 (d, *J* = 4.1 Hz, 1H), 4.57 (td, *J* = 9.1, 3.5 Hz, 1H), 2.04–1.86 (m, 1H), 1.73–1.62 (m, 1H), 1.62–1.49 (m, 1H), 1.37–1.18 (m, 2H), 1.10–0.97 (m, 1H), 0.55 (d, *J* = 3.9 Hz, 3H) and 0.46 (d, *J* = 3.8 Hz, 3H); **^13^C NMR (101 MHz, D_2_SO_4_)**, δ 153.2, 152.3, 151.5, 147.3, 115.2, 66.4, 51.3, 43.9, 43.6, 36.6, 34.5, and 33.5.

## Figures and Tables

**Table 1:** Summary results of the stability tests of the selected cyclopentyl nucleosides in 98 wt% D_2_SO_4_, 2% D_2_O at RT ^1^. Color shading corresponds to the degree of compound degradation in concentrated sulfuric acid. No degradation (dark green), minor degradation (light green), large degradation (light pink), complete degradation (dark pink).

	Structure	Inc. Time: 1–2 h	Inc. Time: 2 d	Inc. Time: 7 d	Inc. Time: 14 d
**A1**	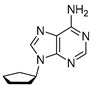	No degradation	No degradation	2.5% degraded to adenine	4% degraded to adenine
**G1**	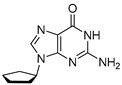	No degradation	No degradation	No degradation	No degradation
**C1**	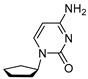	Fully degraded to cytosine	Fully degraded to cytosine	Fully degraded to cytosine	Fully degraded to cytosine
**T1**	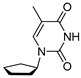	No degradation	No degradation	No degradation	No degradation
**A2**	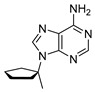	Fully degraded to adenine	Fully degraded to adenine	Fully degraded to adenine	Fully degraded to adenine
**A3**	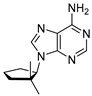	13% degraded to adenine	20% degraded to adenine	33% degraded to adenine	48% degraded to adenine
**A4**	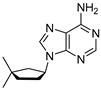	No degradation	No degradation	No degradation	No degradation

^1^ Reaction conditions: 14–16 mg of cyclopentyl nucleosides were dissolved in 0.60 mL 98% *w*/*w* D_2_SO_4_ (2 wt% D_2_O), followed by dilution with 0.06 mL DMSO-*d_6_*. NMR spectra were measured after 1–2 h, 2 days, 7 days and 14 days. The estimate of the percentage of degradation has been calculated by integrating the area below the key H peaks on the ^1^H NMR spectra.

## Data Availability

All data needed to evaluate the conclusions in the paper are present in the paper and available for download from Zenodo at https://zenodo.org/records/18441685.

## Abbreviations

The following abbreviations are used in this manuscript.

NMR	Nuclear magnetic resonance spectroscopy
RT	Room temperature
